# A novel targeted lung denervation multi-polar radiofrequency ablation system for moderate to severe COPD patients: a translational study

**DOI:** 10.1186/s12931-026-03496-7

**Published:** 2026-01-13

**Authors:** Rui Xu, Liangyuan Li, Yongchun Shen, Xiaoju Tang, Hui Zhu, Kaige Wang, Hong Xu, Liheng Xie, Chenhui Su, Yuying Jiang, Dan Liu, Fengming Luo

**Affiliations:** 1 Department of Pulmonary and Critical Care Medicine, West China Hospital, Sichuan University, 37 Guoxue Lane, Wuhou District, Chengdu, Sichuan Province 610041 China; 2 Hangzhou Broncus Medical Co., Ltd., Hangzhou, Zhejiang Province China

**Keywords:** Chronic obstructive pulmonary disease, Targeted lung denervation, Anticholinergic, Multi-polar radiofrequency ablation

## Abstract

**Background:**

Disruption of parasympathetic pulmonary nerves, which release acetylcholine and trigger airway smooth muscle constriction, has been shown to improve lung function and alleviate symptoms in patients with chronic obstructive pulmonary disease (COPD). However, the current targeted lung denervation (TLD) mono-polar radiofrequency (RF) ablation system has the potential for structural improvement to enhance the generalizability and safety of the TLD procedure.

**Objective:**

To develop a novel TLD multi-polar RF ablation for COPD treatment and evaluate its feasibility, safety, and efficacy.

**Methods:**

In the preclinical study, we performed TLD in vitro (porcine lung and liver model) to validate its feasibility and in vivo (dogs and sheep) to ensure its safety and preliminary efficacy. Subsequently, we conducted a first-in-man study to evaluate TLD in patients with COPD forced expiratory volume in 1 s (FEV_1_)/forced vital capacity (FVC) (FEV_1_/FVC < 0.70; FEV_1_ 20%–60% predicted) with three energy settings (12 W, 14 W, and 16 W). The primary safety endpoint was the occurrence of any adverse events or serious adverse events deemed related to the TLD device or the procedure. The efficacy endpoints included the instrument and technical success rates of the TLD procedures, as well as changes in lung function, exercise capacity assessments, and health-related quality of life.

**Results:**

In vitro experiments demonstrated that using ice-cold saline irrigation reduced the temperature at the ablation point compared to room-temperature saline (44 °C vs. 63 °C). The ablation range was 6–8 mm when the single electrode power was 12–16 W, coinciding with the distribution of peribronchial nerves. In the in vivo experiments, we confirmed the feasibility of performing TLD in dogs without causing esophageal injury. In sheep, the bronchoscopy and histological examinations showed airway epithelial restitution within a one-year follow-up. Postprocedural pulmonary airway resistance was reduced by approximately 30% with a sustained 30% decrease in axonal staining. In the first-in-man study, the nine patients included reported good tolerance with a success instrument rate of 100% and a technical success rate of 88.9%. FEV_1_ increased by 160 ± 120 mL at 6 months post-TLD and 80 ± 150 mL at 12 months post-TLD from baseline. The patients’ motor ability and quality of life scores showed improvement but returned to baseline levels by the twelfth month.

**Conclusion:**

This study demonstrated the feasibility of the novel TLD multi-polar RF ablation system in COPD patients. Its safety and clinical efficacy require further validation in larger patient cohorts.

**Clinical trial number:**

ChiCTR2100047843 (http://www.chictr.org.cn/). Registration date: 27 June, 2021.

**Supplementary Information:**

The online version contains supplementary material available at 10.1186/s12931-026-03496-7.

## Introduction

Patients with moderate and severe chronic obstructive pulmonary disease (COPD) continue to suffer from unresolved symptoms of breathlessness, activity limitation, and risk for exacerbation despite optimal medical treatment [1, 2]. The pathophysiology of COPD is characterized by increased autonomic nervous system input to the lungs and sensory signaling from the lungs mediated by the vagal nerve [3]. Increased parasympathetic input raises cholinergic tone in the airways, which modulates airway smooth muscle tone, airway hyperresponsiveness, inflammation and mucus hypersecretion [3, 4]. Pharmacologic inhibition of parasympathetic input using inhaled anticholinergic agents has long been a main COPD treatment and has demonstrated efficacy in reducing symptoms and exacerbations [5]. However, drug deposition is often limited in poorly ventilated regions of the lung, particularly during exacerbations, resulting in suboptimal disease control in a substantial proportion of patients [6–8]. Surgical denervation of pulmonary vagal inputs remains clinically impractical due to its invasiveness and associated risks [9].

To address these limitations, targeted lung denervation (TLD) has emerged as a promising bronchoscopic approach that aims to disrupt parasympathetic nerve trunks surrounding the main bronchi using radiofrequency (RF) ablation [10]. Early clinical studies have demonstrated the safety and feasibility of this technique, with evidence of improved airway function and symptom control [11–13]. TLD is typically performed via a balloon catheter equipped with an internal cooling system to prevent thermal injury to the airway mucosa while delivering energy at sufficient depth to ablate vagal nerve fibers [10]. However, the current TLD ablation system faces several challenges [10, 14, 15]. First, the mono-electrode design requires multiple rotational activations within each bronchi to achieve circumferential ablation and may result in multiple ablations at the same site. Second, the closed-loop internal coolant system lacks real-time thermal regulation, raising the risk of localized overheating and tissue injury when balloon contact with the airway is suboptimal. Third, the absence of an adjustable ablation tip limits adaptability to interindividual variations in airway anatomy.

## Methods

### Composition and TLD procedure of the novel system

**Fig. 1:**
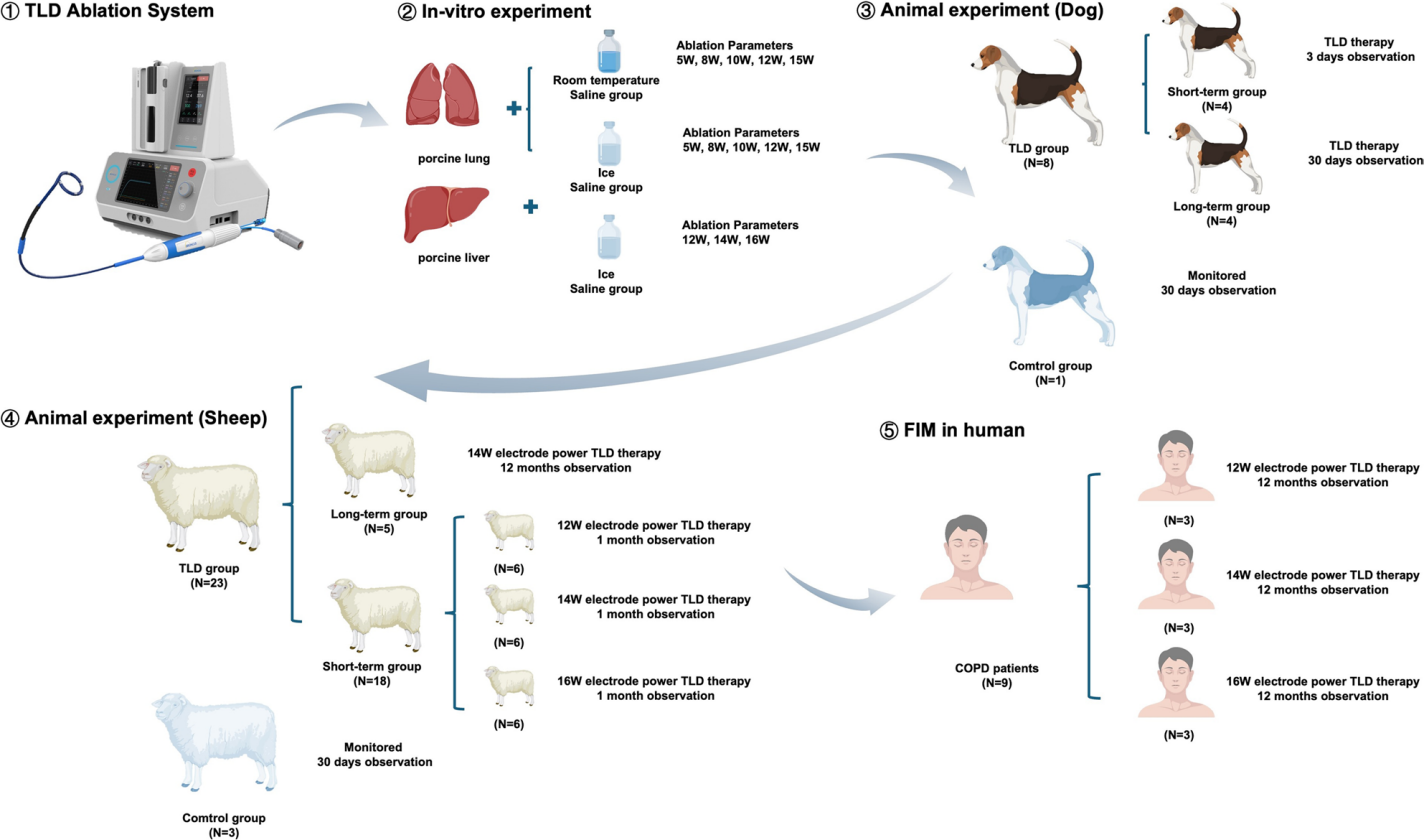
The flowchart of the overall study design. Generic Diagramming Platform (https://biogdp.com/) was used to draw schematic diagrams

**Fig. 2:**
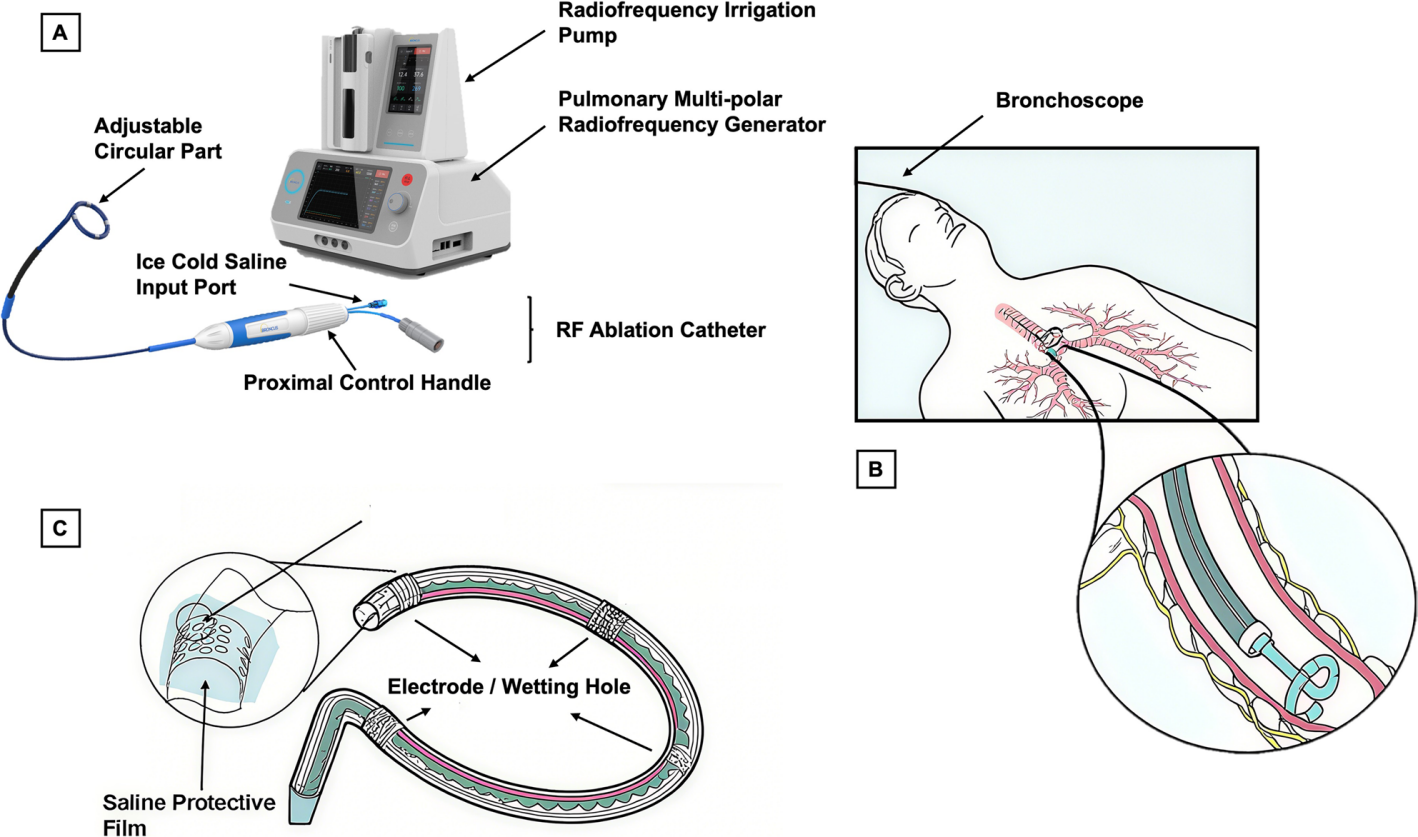
Targeted Lung Denervation Radiofrequency Ablation System. **A** The composition of the novel system. **B** The TLD ablation catheter reaches the target airway for ablation through bronchoscopy. **C** The detail of the adjustable circular ablation catheter tip

The overall study design is shown in Fig. 1. The novel targeted lung denervation radiofrequency ablation system (BroncAblate; Hangzhou Broncus Medical Co., Ltd., China) consists of a pulmonary multi-polar radiofrequency generator, a disposable radiofrequency ablation catheter, and a radiofrequency irrigation pump (Fig. 2A). The multi-polar RF generator delivers RF energy via a multi-electrode discharge mode; the disposable RF ablation catheter is designed for bronchoscopic access (Fig. 2B) and transmits RF energy to the airway to achieve targeted ablation of nerves; the RF irrigation pump delivers sterile saline through the catheter lumen to the electrode surface, enabling dynamic regulation of tissue temperature and impedance to prevent eschar formation at the target site. The catheter tip (Fig. 2C) with four equidistantly distributed electrodes is designed as an adjustable circular part, which allows passive contraction to conform to the airway lumen. The operator can also actively adjust the loop diameter through a proximal control handle, enabling fine-tuning to optimize contact between the electrodes and the airway wall.

The TLD procedure is performed sequentially in both main bronchi in a single bronchoscopic session under general anesthesia. First, ablation parameters are preset on the RF ablation system. Sterile saline is then injected through the catheter via the irrigation pump to expel air from the saline channel. The catheter (1.9 mm-diameter) is advanced through the sheath in the bronchoscope’s working channel (≥ 2.8 mm-diameter) to the target site. The ablation site is located at the opening of the left main bronchi or the right main bronchi, approximately 1–2 cm from the carina. The circular part of the ablation catheter tip is actively adjusted via a control knob on a proximal control handle to ensure adequate contact of electrode and airway wall. The saline (20–30 mL, each side) is delivered via the RF irrigation pump, which pushes the fluid from the syringe through the extension tubing and internal liquid channel of the catheter to the electrode outlets. Once saline flow at the distal tip is confirmed, RF energy is delivered according to the predefined parameters to complete target ablation. During the whole procedure, the RF generator continuously displays the real-time impedance and temperature of each electrode. Finally, catheter withdrawal is performed under bronchoscopic visualization after confirmation of safety.

### In-vitro experiment on porcine lung and liver models

An in vitro porcine lung model was used to investigate the protective effect of saline irrigation on bronchial tissues during the TLD procedure. Fresh porcine lungs were obtained, and a longitudinal incision was made to access bronchial structures of suitable diameter. Appropriate ablation sites were selected based on bronchial size and anatomical accessibility. An RF ablation catheter was positioned at the predefined target site, and the impedance was monitored on the RF generator. The position of the neutral electrode was adjusted relative to the lung tissue to approximate the baseline impedance typically observed in human lungs. RF ablation was conducted using five single electrode power settings: 5 watts (W), 8 W, 10 W, 12 W, and 15 W. Saline irrigation was delivered through the catheter at a constant rate of 10 mL/min under two temperature conditions: room temperature (~ 25 °C) and ice-cold (0–5 °C). For each ablation point, the RF irrigation pump was activated to initiate saline irrigation, and RF energy delivery was started once saline outflow from the catheter tip was observed. Throughout the ablation process, temperature probes were used to monitor and record the local tissue temperature at the ablation site and the adjacent tissue.

An in vitro porcine liver model was used to investigate the ablation effect of the TLD procedure. The ablation site is selected on the surface of the porcine liver, and the electrode is connected to the ablation site with saline to form a circuit. The impedance adjustment and ablation method were performed the same as in porcine lungs, with a constant rate of 15 mL/min of ice-cold saline irrigation during the procedure. After ablation was completed, the tissue was dissected along the sagittal plane at the ablation site, and the depth of ablation was measured at different single electrode power settings (12 W, 14 W, and 16 W).

### In vivo experiment on dogs and sheep

#### Study design of the animal study

A pilot experiment was designed to initially explore the in vivo safety of TLD in eight experimental dogs and one control dog with single electrode powers ranging from 12 W to 16 W. Four dogs in the experimental group were euthanized 3 days after the TLD procedure, and the other four dogs were monitored for one month.

In the formal experiment, short-term and long-term experiments were conducted on sheep. In the short-term experiment, 18 sheep were treated with a single electrode of different energies: 12 W (*n* = 6), 14 W (*n* = 6), and 16 W (*n* = 6), with three control sheep, and were followed up at 7-day intervals after TLD procedures and euthanized after one month. Based on the results of the short-term experiment, an optimal single electrode power energy (14 W) was selected and applied to 5 sheep in the long-term experiment, which was monitored and evaluated at 3-month intervals until they were euthanized 12 months after TLD procedures to assess the long-term safety and efficacy.

This study was approved by the Ethical Committee of WestPoint Innovation Center (Ethical Approval number: IAC21110101, IAC22041801), and treatment of the animals was in accordance with the Animal Welfare Act of 1966 and all efforts were made to minimize animal suffering and to use the minimum number of animals necessary to produce reliable scientific data.

#### Airway resistance measurement

To confirm the sensitivity of airway resistance measurements to bronchodilation, all sheep underwent an atropine challenge test two weeks before baseline assessment. Atropine (0.15 mg/kg) was administered intravenously after an initial lung resistance measurement, and airway resistance was recorded every minute thereafter. Formal airway resistance measurements were conducted using the EMMS forced oscillometry system at baseline, and at 14 days, as well as 1, 3, 6, 9, and 12 months post-TLD. During all assessments, animals were anesthetized, and spontaneous breathing was suppressed. Ventilation was fully controlled using a ventilator set at 15 breaths per minute with a tidal volume of 500 mL.

#### Histological and immunohistochemical assessment of bronchial structure and lung denervation effect

Animals were euthanized following bronchoscopy at the end of the designated follow-up period. The chest cavity was opened and the heart, pericardium, aorta, esophagus, mediastinum, and lungs were evaluated grossly. Any observed gross lesions were trimmed for processing and evaluation. Histological changes at the ablation sites (including the bronchial mucosa (epithelium and lamina propria), submucosa, smooth muscle, cartilage, and adjacent lung parenchyma) were evaluated on continuous sections for each bronchus via HE staining. Axonal staining of the selected airway was performed using immunohistochemical staining with Pan Neuronal Marker (pNM) (1:200 dilution, Millipore Corp, Billerica, Mass, USA), and a semiquantitative analysis of pNM gray value corrected to the axonal area was conducted with HALO software (v3.3.2541.424).

### First-in-man study

#### Study design and participants

Based on preclinical studies confirming the safety and efficacy of these three energy levels, and considering the anatomical and physiological differences between animals and humans, we designed a non-randomized, prospective, dose-escalation first-in-man (FIM) study, which was conducted between August 2021 and June 2023 in West China Hospital of Sichuan University. The study is designed to begin with the enrollment of subjects in the 12 W group, and bronchoscopic examination is evaluated at the one month post-TLD follow-up. If there was no significant localized damage to the tracheal mucosa and the subjects tolerated the energy treatment well, we proceeded to the screening and enrollment session of patients in the 14 W group. The screening and enrollment session for patients in the medium energy treatment group to enter the 16 W group was the same.

Based on published literatures [13–15], eligible patients were ≥ 40 years of age with COPD, defined as the ratio of the forced expiratory volume in 1 s (FEV_1_) to the forced vital capacity (FVC) of ≤ 0.70 and postbronchodilator FEV_1_ of 20%–60% of predicted normal values, and documented a history of taking inhaled drugs as routine respiratory maintenance medications for ≥ 12 months at the time of informed consent. The full list of inclusion and exclusion criteria was presented in Supplementary Table 1.

This study was approved by Ethical Committee of West China Hospital of Sichuan University (Ethical Approval number: 2021 − 426) and in accordance with the Declaration of Helsinki (1996), Good Clinical Practice guidelines, and local requirements. An operations committee, and a data monitoring committee, oversaw protocol management and safety for the study. An independent clinical event reviewer adjudicated all serious adverse events (SAE). This trial is registered with Chinese Clinical Trial Registry, number ChiCTR2100047843.

#### Study endpoints and procedures

The study’s primary safety endpoint is the occurrence of any adverse events (AEs) or serious adverse events (SAEs) attributable to the TLD device or procedure within 6 months post-TLD. The primary efficacy endpoints include the following two success rates: (1) instrument success, defined as the ability to place the ablation catheter at the ablation site and withdraw after TLD procedure; (2) technical success, defined as the ability to deliver RF energy to the expected ablation site and complete the treatment. The secondary endpoints included change from baseline in lung function tests, exercise capacity assessments, and health-related quality of life (QoL).

After informed consent, a wash-out period of 8 days for long-acting muscarinic antagonists (LAMA), 24 h for long-acting β agonists (LABA) and 12 h for short-acting β agonists (SABA) and short-acting muscarinic antagonists (SAMA). Patients underwent baseline and follow-up investigations including standard spirometry, chest computed tomography scan, 6 min walk test (6MWT), COPD-specific St. George’s Respiratory Questionnaire (SGRQ-C; score range 0–100 with a minimally clinically important difference (MCID) of ≥ 4 negative units), COPD assessment test (CAT, score range 0–40), modified medical research council scales (mMRC, score range 0–4) and Gastroparesis Cardinal Symptom Index Questionnaire (GCSI, score range 0–45).

After completion of the wash-out baseline testing, patients underwent a minimum 7-day run-in period while on tiotropium bromide, and similar testing was performed at drug trough 24 h after the last dose of tiotropium to establish tiotropium trough baseline values. On-drug testing was repeated by hospital visits at 1, 3, 6, 9, and 12 months after the TLD procedure. A total of two bronchoscopies were performed on each patient, the first to complete the TLD treatment and the second to assess airway condition on the 1-month follow-up visit. Adverse events were tracked and recorded throughout the entire study period.

### Statistical analysis

For in vitro and dog experiments, samples and data collected preoperatively, intraoperatively, and postoperatively were recorded in detail and analyzed descriptively, without statistical data analysis. For the experiments in sheep, the samples and data collected at preoperative, intraoperative, and postoperative follow-up were recorded in detail with descriptive analyses and statistical analyses. T-tests or Mann-Whitney U-tests were performed according to the collected samples and data. For the first-in-man study, there was no primary study hypothesis with statistical inference because of the primary exploratory nature. Continuous data were summarized using means and SDs, or range, in the presence of non-normality. Categorical data were tabulated, with counts and percentages. ANOVA tests were used to assess the statistical differenced of changes of lung function between the different time points and the baseline. All statistical analyses were performed with GraphPad Prism 10 (GraphPad Software, San Diego, CA, USA) and SPSS version 25 (SPSS Inc., Chicago, IL, USA). *P* < 0.05 was considered statistically significant.

## Results

### In vitro experiment

**Fig. 3:**
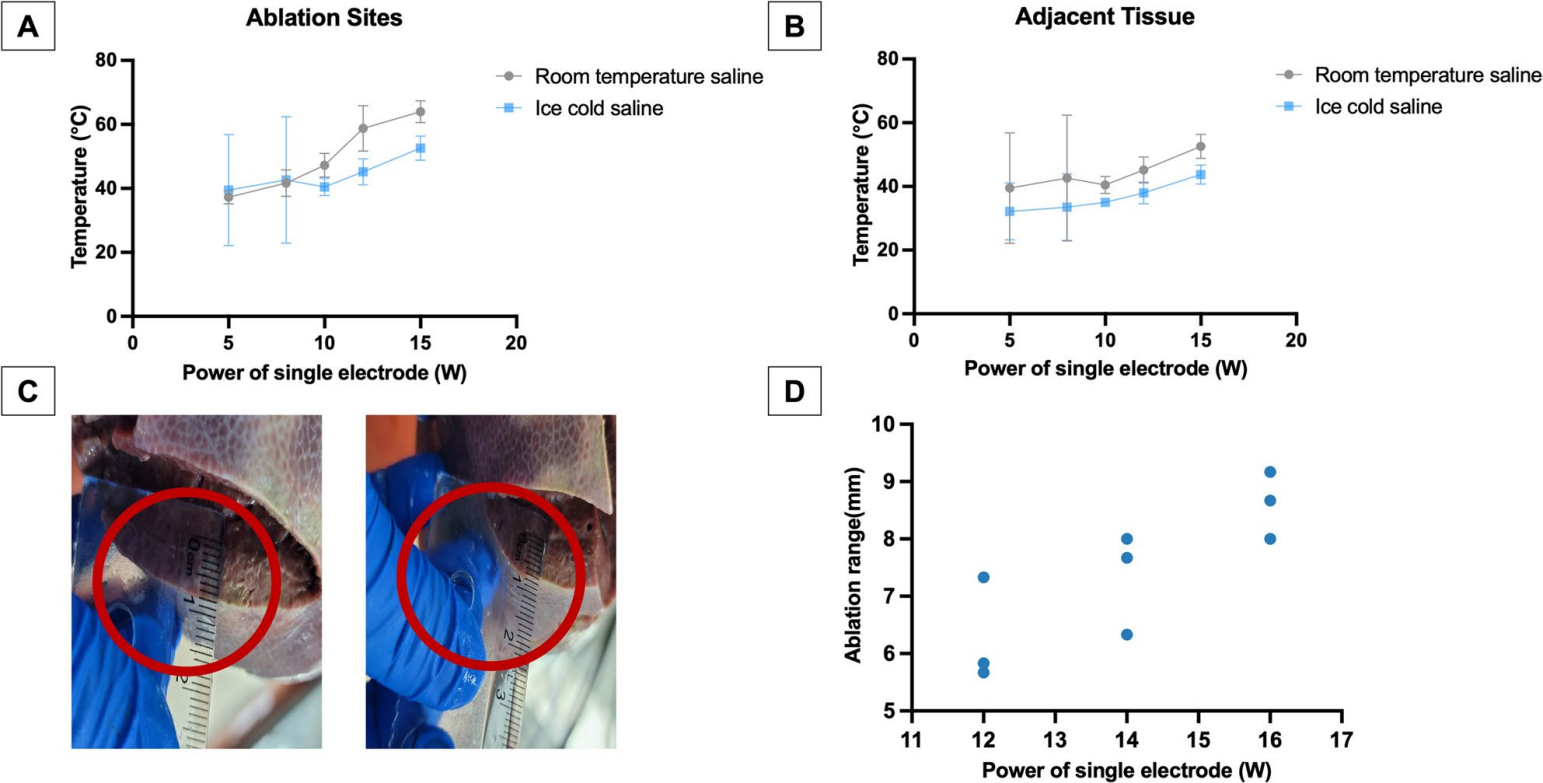
Results of the in vitro experiments. Temperature of (**A**) ablation sites, (**B**) adjacent tissues during TLD procedures under different ablation powers and different saline temperatures in the porcine lung model. **C** The ablation range measured after the liver was cut. **D** Average ablation range under different single electrode powers in the porcine liver model

During the ablation in the porcine lung model, we observed that when the single electrode power was increased from 5 to 15 W, the temperature at the ablation point using room-temperature saline perfusion rose to 63 °C. In contrast, when using ice-cold saline irrigation, the temperature at the ablation point was only 44 °C (Fig. 3A), and the temperature of the adjacent tissues was controlled within an acceptable range (Fig. 3B). This suggests that the ice-cold saline irrigation system significantly reduced the temperature at the ablation point compared to room-temperature saline and there was no significant difference in the temperature of the adjacent tissues.

Based on the ice-cold saline irrigation being effective in avoiding tissue damage caused by high temperatures during ablation, we continued to explore the optimal single-electrode power for ablation in the porcine liver model (Fig. 3C). Results showed the ablation range was 6–8 mm when the single electrode power was 12–16 W (Supplementary Tables 2 and Fig. 3D), which was considered adequate to achieve the depth for denervation.

### TLD procedure on dogs

**Fig. 4:**
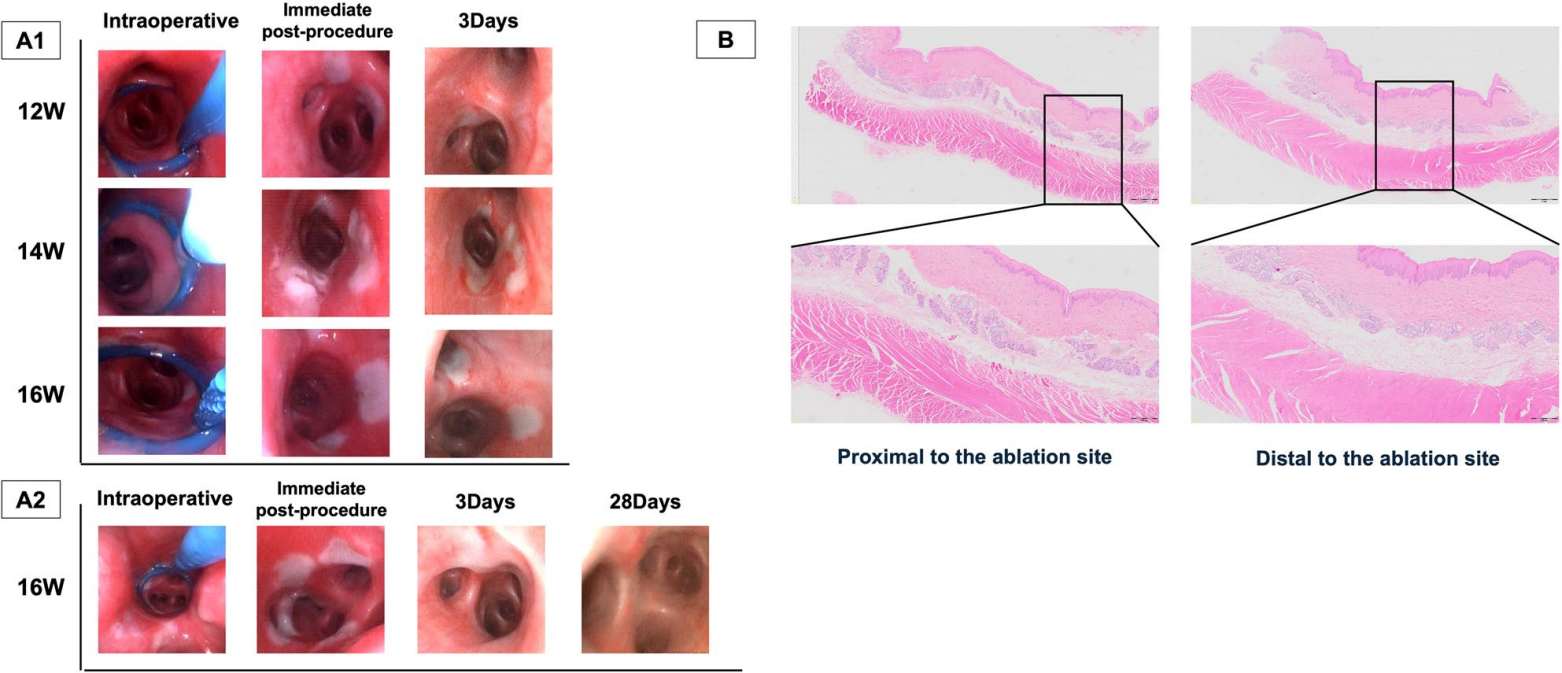
Representative bronchoscopic images of bronchi and HE staining of esophageal tissues in dogs. **A1** Bronchoscopic findings in short-term groups during TLD procedure and follow-up. Airway discoloration occurred immediately postoperatively and at 3 days in all power groups. **A2** Bronchoscopic findings in long-term groups during TLD procedure and follow-up. At 28 days, all main bronchi appeared grossly normal even at maximum power (16 W), with occasional mild hyperemia. **B** Esophageal tissues proximal and distal to ablation sites. No histopathological damage was observed in periprocedural esophageal tissues post-TLD

The TLD procedure achieved accurate arrival at the ablation site, smooth deployment of the catheter coil, successful saline injection, and sustained release of ablation energy in the eight dogs. After TLD treatment, local asymptomatic airway blanching of the eight experimental dogs was immediately observed (Fig. 4A1). In the 3-day observation group, there was one case of slight abrasion of the esophagus in an experimental dog, and samples were taken and stained with HE, which showed no obvious histological features such as necrosis, inflammation, or fibrous damage (Fig. 4B). In the 28 days observation group, the appearance of the airway was essentially normal with mild congestion at 28 days post-TLD (Fig. 4A2). One dog developed a postprocedural cough, which resolved one day later without any treatment. No adverse events or significant gross lesions in the lungs, heart, or pericardium of the experimental dogs were observed during the follow-up period. Therefore, we conclude that a single electrode power of 12–16 W is safe in animals.

### TLD procedure on sheep

#### Feasibility and safety

The TLD procedure achieved accurate arrival at the ablation site, smooth deployment of the catheter coil, successful saline injection, and sustained release of ablation energy in all sheep in the short and long-term groups. In the short-term group, the ablation process took 365.61 ± 28.61 s in total, without difference between the left and right bronchi (185.72 ± 23.72 s for the left bronchi, and 179.88 ± 20.20 s for the right bronchi, *p* > 0.1). In the long-term group, the ablation process took 332.4 ± 31.9 s in total, without difference between the left and right bronchi (179.4 ± 30.6 s for the left bronchus, and 153.0 ± 12.6 s for the right bronchi, *p* > 0.05). In the short-term group, two sheep developed arrhythmia before or after the TLD procedure, and two sheep experienced cough after the TLD procedure; these symptoms resolved within two days. No adverse events occurred in the long-term group. The results of routine blood tests and liver and renal function tests in experimental sheep before and after TLD procedures showed no abnormalities (Supplementary Table 3).

Bronchoscopic evaluation of the airway wall revealed immediate post-TLD alterations characterized by acute blanching and congestion in the airway mucosa contacting the ablation site. In the short-term group (Supplementary Fig. 1A1), a minority of sheep (4/18) exhibited smooth mucosal surfaces at the 14-day assessment, while the predominant findings (14/18) included either persistent whitish mucosal lesions indicative of tissue repair or granulomatous changes with proliferative stroma, histologically confirmed as non-caseating inflammatory responses. Focal endobronchial mucus adhesion was concurrently observed in sporadic cases (6/18). By 28 days post-TLD, progressive mucosal restoration became evident in more sheep (11/18), with residual lesions displaying active resolution through nascent re-epithelialization; mucus adhesion had resolved in 77.8% of subjects (14/18). In the 14 W long-term group evaluated at 12 months (Supplementary Fig. 1A2), complete histological resolution of bronchial wall lesions occurred universally, culminating in restoration of near-normal epithelial architecture.

Histological analysis of ablation sites via H&E staining revealed epithelial denudation, goblet cell hyperplasia, and inflammatory infiltrates within the lamina propria (Supplementary Fig. 1A1). These pathological changes exhibited energy-dependent severity escalation, yet crucially, no epithelial necrosis or fibrosis was identified. Notably, focal interstitial fibrosis developed in adjacent alveolar parenchyma in a subset of 16 W group animals. By 3 months post-TLD in the 14 W long-term group (Supplementary Fig. 1A2), near-complete tissue restitution had occurred with minimal residual granulation tissue. A single animal developed persistent hypertrophic scarring at a unilateral tracheal ablation site, which remained histologically evident at 12-month follow-up.

#### Evaluation of airway resistance

**Fig. 5:**
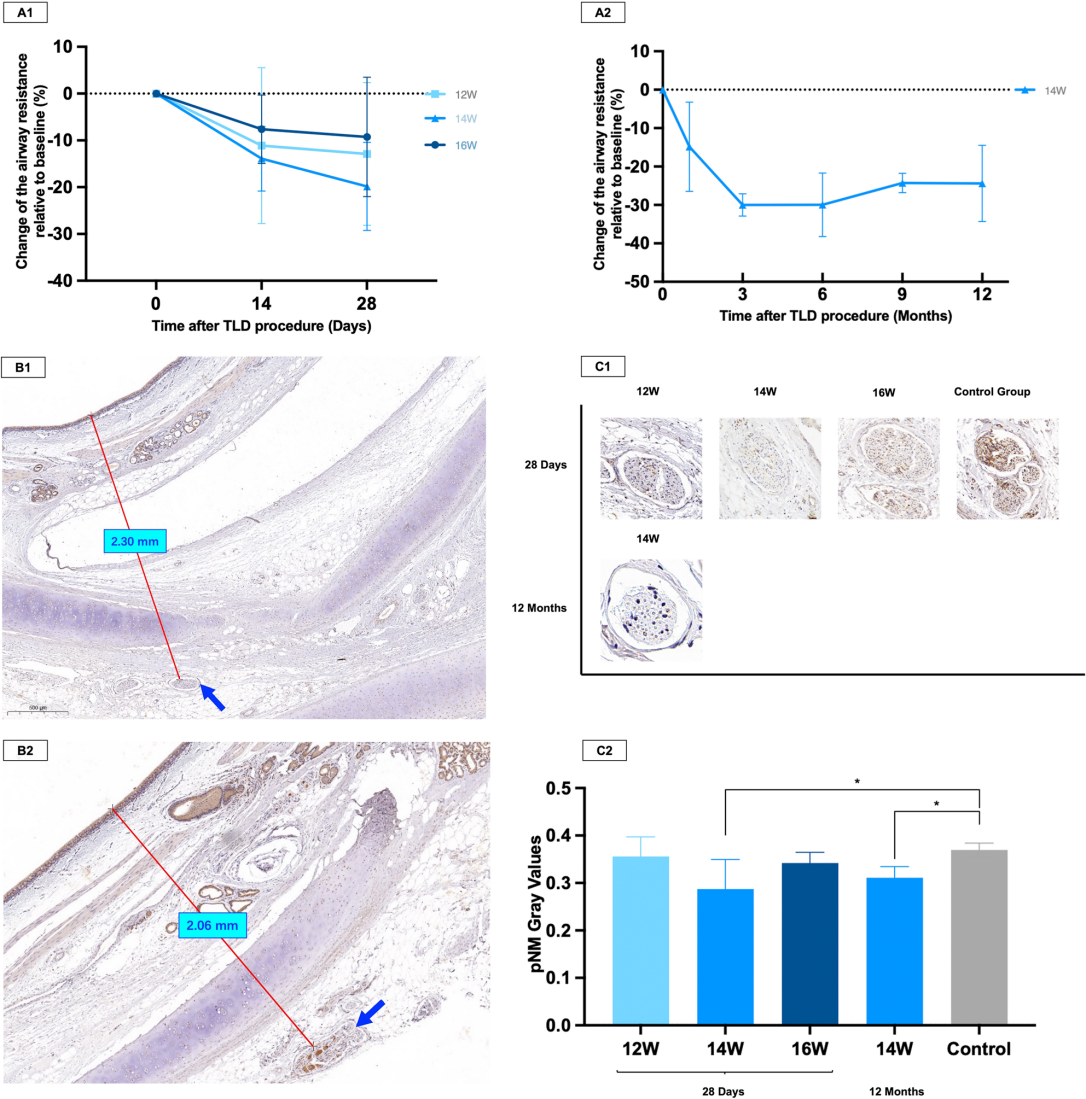
Airway resistance test and pNM staining results in sheep. (**A1**) Relative change in airway resistance from baseline at 28 days post-TLD across power settings. (**A2**) Relative change in airway resistance from baseline at 12 months post-14 W TLD. pNM staining showed reduced neural staining in the 14 W short-term group (**B1**) than the control group (**B2**). The solid line indicates the distance from the airway epithelium to the nerve bundles, and the blue arrows point to the neurons. (**C1**) Representative pNM staining in 12 W/14 W/16 W treatment groups and 14 W group at 28 days/12 months. (**C2**) Significantly reduced pNM grayscale values in all energy groups compared to the control group (*P* < 0.05 only for 14 W group)

All sheep’ atropine tests before TLD procedures were positive. Figure 5A1 shows the change in the airway resistance after TLD procedures. There was a noticeable decrease in airway resistance at 14 days post-TLD, which stabilized until 28 days post-TLD. Animals in the 14 W group showed the greatest reduction in airway resistance (decreased 13.92%±6.90% at 14 days post-TLD and 19.84%±9.42% at 28 days post-TLD compared to baseline).

In the long-term group (14 W), the lowest airway resistance was recorded at the 3 months post-TLD (decreased 29.97%±2.91% compared to baseline) and remained low until the 9 months post-TLD. One year after TLD procedures, there was a 24.39%±9.92% reduction in airway resistance compared to the baseline, and the difference is statistically significant, as shown in Fig. 5A2.

#### Histological and immunohistochemical assessment of and the denervation effect

H&E staining of sheep bronchial tissue revealed persistent neuronal vacuolation and pyknotic nuclei at ablation sites acutely and at 1-month post-TLD (Supplementary Fig. 1B1), indicating axonal degeneration consistent with neuroablative efficacy. pNM immunohistochemistry demonstrated significantly reduced axonal expression in peribronchial nerves across all power cohorts versus controls (Fig. 5B and C), with maximal denervation at 14 W (*p* = 0.03). Therefore, a single electrode ablation power of 14 W was chosen in the long-term study.

In the long-term study, diminished but persistent neuronal vacuolation and nuclear pyknosis were observed one year after TLD procedures (Supplementary Fig. 1B2). The denervation effects persisted for a full year following TLD procedures as indicated by the pNM staining gray levels (Fig. 5C). Crucially, circumferential collagenous deposition within the bronchial adventitia formed organized fibrotic bands with Masson staining (Supplementary Fig. 1B3-1B4), which was supposed to prevent axonal regrowth. Therefore, we conclude that TLD achieved durable (> 12-month) denervation.

### Clinical results

#### Baseline characteristics of the included patients

**Fig. 6:**
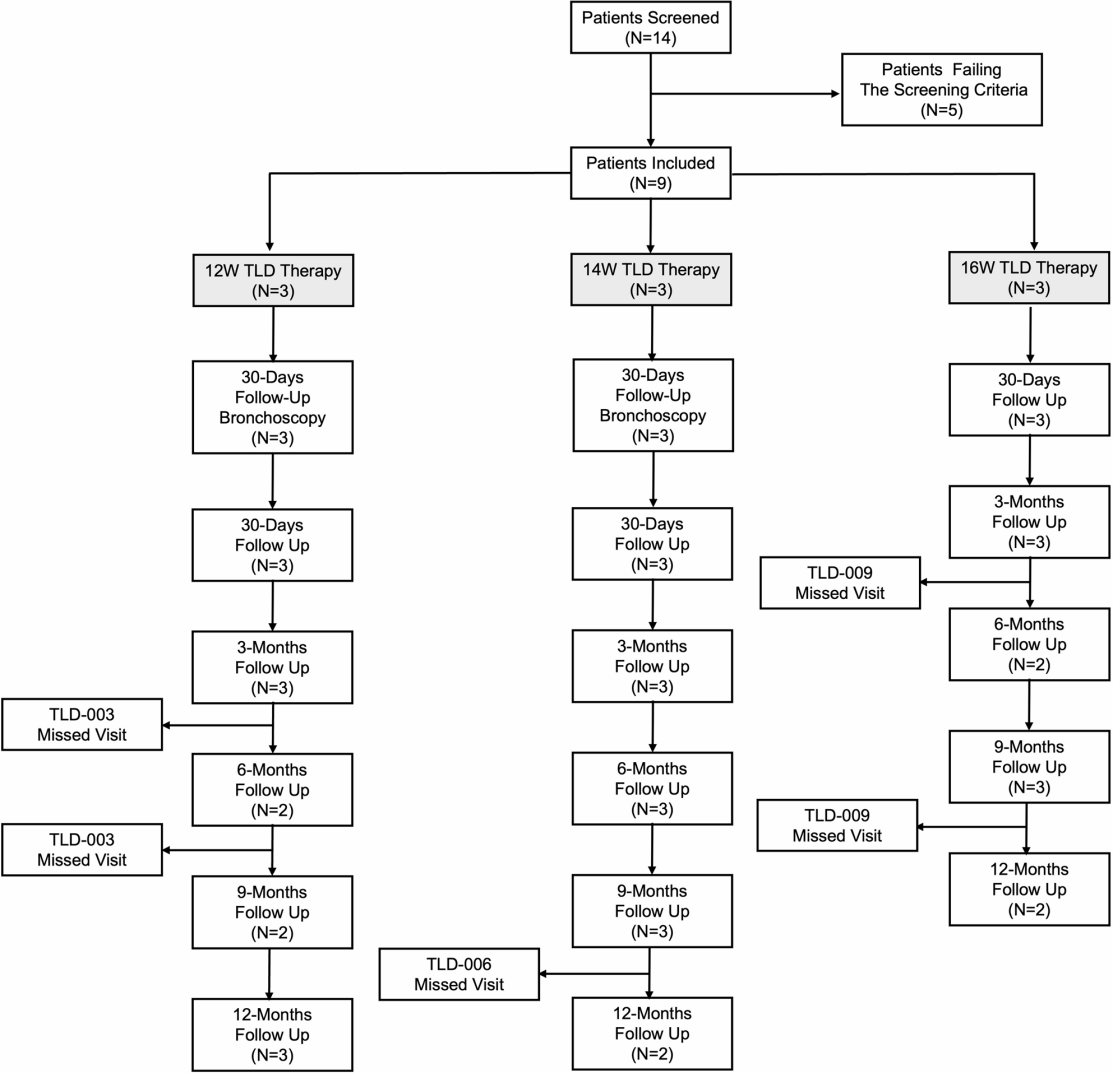
Flow chart of the first-in-man study

A total of nine patients were included in the first-in-man study (Fig. 6), and the baseline characteristics are summarized in Table 1. During the 6-month follow-up period, Patient No.3 missed the 6- and 9-month follow-up visits, Patient No.6 missed the 12-month follow-up, and Patient No.9 missed the 6-month follow-up. The reasons for loss to follow-up were acute exacerbation of chronic obstructive pulmonary disease or COVID-19 infection.

**Table 1:** Baseline characteristics of included patients

Items	Total
Demographic information	
Age (years)	67 ± 7
BMI index (kg/m^2^)	22.8 ± 3.9
Smoking history (pack years)	27.50 ± 11.32
Lung fuction	
FEV_1_(L)	0.77 ± 0.12
FEV_1_% Pred(%)	29.77 ± 4.90
FVC(L)	2.07 ± 0.25
FVC% Pred(%)	62.82 ± 7.86
FEV_1_/FVC(%)	37.47 ± 5.68
TLC(L)	7.66 ± 1.69
TLC% Pred (%)	128.38 ± 25.92
RV(L)	5.60 ± 1.68
RV% Pred (%)	237.19 ± 72.56
FRC(L)	6.95 ± 1.70
Scales and test	
mMRC score	2.22 ± 0.66
CAT score	17.89 ± 5.16
SGRQ-C score	46.36 ± 12.55
6MWD (m)	319 ± 84.98
COPD medication (*n*, %)	
LAMA	1(11.1)
LABA + ICS*	4(44.4)
LAMA + LABA + ICS	4(44.4)
Comorbidities (*n*, %)	
Hypertension	3(33.3)
Diabetes	3(33.3)
Cor pulmonale	2(22.2)
Chset CT features	
Airway measurement	
Right main bronchi length (mm)	19.80 ± 3.94
Right main bronchi average diameter (mm)	15.07 ± 2.81
Left main bronchi length (mm)	39.60 ± 4.03
Left main bronchi average diameter (mm)	13.86 ± 1.69
Goddard score	10.67 ± 4.06
Mucus plug score	0.67 ± 0.86
Reffi score	2.40 ± 2.79

*These patients were on LAMA treatment one year before baseline but without satisfactory disease control and received LABA + ICS at the time of consent. *BMI* Body mass index, *mMRC* Multiple myeloma research consortium, *CAT* COPD assessment test, *SGRQ* Saint George’s respiratory questionnaire, *6MWD* 6 min walking distance, *LAMA* Long-acting anticholinergic agents, *LABA* Long-acting β2 agonists, *ICS* Inhaled corticosteroid, *N* Total number of people

### The feasibility and safety of the TLD procedure

As for the feasibility, all nine patients completed TLD treatment with an average ablation time of 321 ± 35 s (range: 253–366 s) and the procedure time of 63 ± 20 min (range: 23–99 min). The ablation catheter reached the left and right main bronchi through bronchoscopy, and it was successfully withdrawn after delivering radiofrequency ablation energy with a success instrument rate of 100% (9/9). The radiofrequency ablation energy was successfully delivered to the expected ablation site in eight patients with a total technical success rate of 88.9% (8/9), because not all energy delivery was accomplished during ablation of the right main bronchus on one patient.

**Fig. 7:**
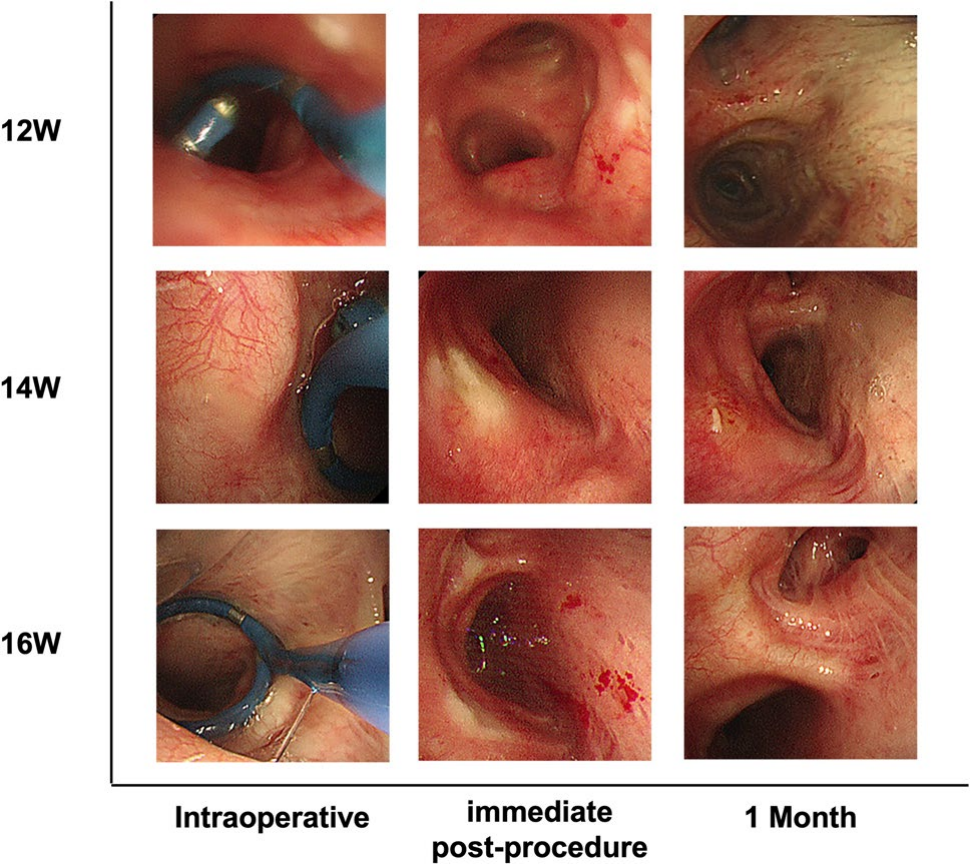
Bronchoscopic findings before and after TLD procedural in humans

As for the safety, the nine patients reported good tolerance and had no complications such as bleeding occurred during TLD procedures. The mean hospital stay was 2.6 ± 0.7 days (range: 1–3 days). During the follow-up period, no deaths and no signs of esophageal nerve damage (such as gastric paresis) were observed. At one month post-TLD, all patients received bronchoscopy examinations. During the bronchoscopy, the bronchi of all nine patients were patent with distinct cartilaginous rings and no bronchial stenosis (Fig. 7); five patients had expected mucosal changes consistent with normal healing; four patients had local ulcers and congestion at the treatment site (all in the 14–16 W groups). Thirteen treatment-related adverse events involving 4 patients (44.4%, 4/9) occurred within 3–6 months following TLD treatment, as shown in Table 2. According to CTCAE V5.0, 84.6% (11/13) of the adverse events are classified as CTCAE level 1 or 2. Summary of treatment-related adverse events within 6 months and within the 12 months after TLD treatment is detailed in Supplementary Tables 4 and Supplementary Table 5. The GCSI score within the 12 months after TLD treatment is shown in Supplementary Table 6.

**Table 2:** Treatment-related adverse events within 3–6 months following TLD treatment

Diagnosis (Patient Could Have Multiple Events)	*n*(%)	Frequency (*n* = 13)
Respiratory tract, thoracic cavity and mediastinum disease	4(44.4)	10
Cough	2(22.2)	2
Expectoration	1(11.1)	1
Dyspnea	1(11.1)	1
Chronic obstructive pulmonary disease *	1(11.1)	2
Upper respiratory tract infection	1(11.1)	2
Chest discomfort	1(11.1)	1
Wheezing	1(11.1)	1
Systemic disease and various reactions at the administration site	1(11.1)	1
Fatigue	1(11.1)	1
Gastrointestinal system disease	1(11.1)	1
Abdominal distension	1(11.1)	1
Musculoskeletal and connective tissue disease	1(11.1)	1
Backache	1(11.1)	1

*Refers to acute exacerbation of chronic obstructive pulmonary disease (AECOPD)

#### The clinical efficacy endpoints

**Fig. 8:**
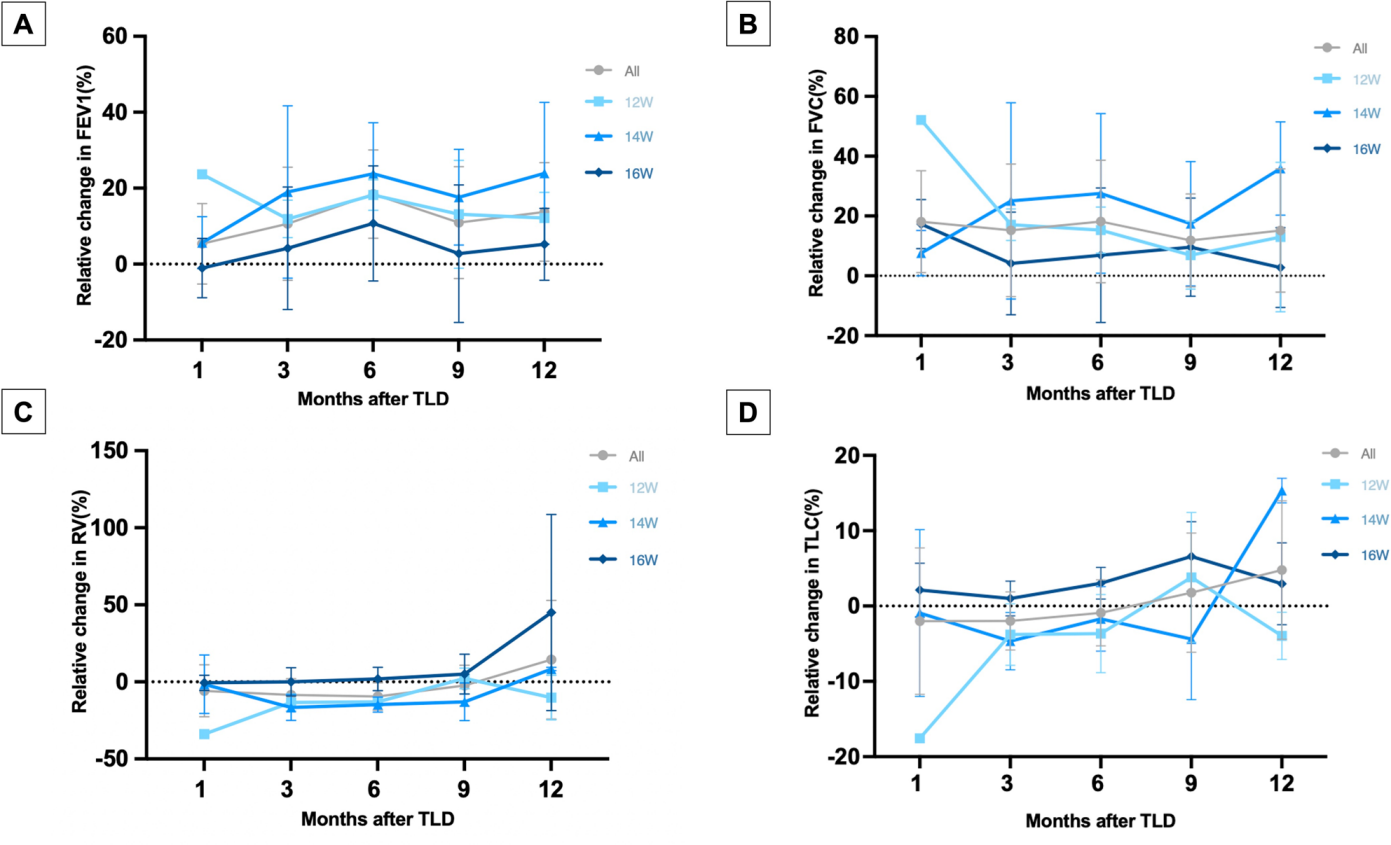
Relative change from baseline in lung function tests FEV_1 _(**A**), FVC (**B**), RV (**C**), TLC (**D**) in each group

As for the primary clinical efficacy endpoints, the improvement in lung function was most pronounced at 6 months following TLD treatment and was sustained through 12 months. FEV_1_ increased by 160 ± 120 mL from baseline at 6 months post-TLD, and 80 ± 150 mL at 12 months post-TLD. Notably, the 14 W group showed the greatest improvement, with a mean increase in FEV_1_ of 200 ± 150 mL at 6 months and 220 ± 200 mL at 12 months. The absolute change results of lung function tests parameters are shown in Table 3. The relative change results of FEV_1_, FVC, RV, and TLC at each time point assessed are shown in Fig. 8.

**Table 3:** Absolute changes of pulmonary function compared to baseline after TLD treatment

	3 months	6 months	9 months	12 months
FEV_1_(L)	0.09 ± 0.13	0.16 ± 0.12^*^	0.10 ± 0.13	0.08 ± 0.15
FEV_1_%pred(%)	3.96 ± 5.41	6.36 ± 4.43^*^	4.20 ± 5.23	3.90 ± 5.51
FVC(L)	0.04 ± 0.37	0.10 ± 0.66	0.12 ± 0.41	0.13 ± 0.55
FEV_1_/FVC(%)	0.50 ± 3.46	0.56 ± 3.71	0.32 ± 4.20	-1.26 ± 5.31
TLC(L)	-0.13 ± 0.29	-0.06 ± 0.32	0.11 ± 0.64	0.21 ± 0.71
TLC%pred(%)	-2.74 ± 6.72	-1.29 ± 7.42	1.65 ± 11.16	6.41 ± 10.95
RV(L)	-0.36 ± 0.46^*^	-0.45 ± 0.42^*^	-0.17 ± 0.83	0.08 ± 0.49
RV%pred(%)	14.13 ± 18.99^*^	18.97 ± 17.80^*^	12.83 ± 36.60	2.74 ± 20.40
IC(L)	0.67 ± 0.65	0.31 ± 0.26^*^	0.38 ± 0.44	0.41 ± 0.40
RAW(kPa*s/L)	-0.09 ± 0.28	-0.19 ± 0.13^*^	-0.07 ± 0.27	-0.25 ± 0.24
sGaw(1/(kPa*s))	0.14 ± 0.27	0.15 ± 0.19	0.12 ± 0.26	0.23 ± 0.34
MEF25%(L/s)	-0.01 ± 0.02	-0.01 ± 0.03	0.00 ± 0.03	-0.03 ± 0.04
MEF50%(L/s)	0.03 ± 0.06	0.06 ± 0.07	0.03 ± 0.10	0.30 ± 0.74
MEF75%(L/s)	0.12 ± 0.25	0.15 ± 0.22	0.13 ± 0.25	0.11 ± 0.27
MEF25-75%(L/s)	0.02 ± 0.05	0.03 ± 0.04	0.02 ± 0.09	0.00 ± 0.07

*Statistically significant compared to baseline, performed in patients with available data during follow-up (3 months: *n* = 8; 6 months: *n* = 7; 9 months: *n* = 8; 12 months: *n* = 7)

**Fig. 9:**
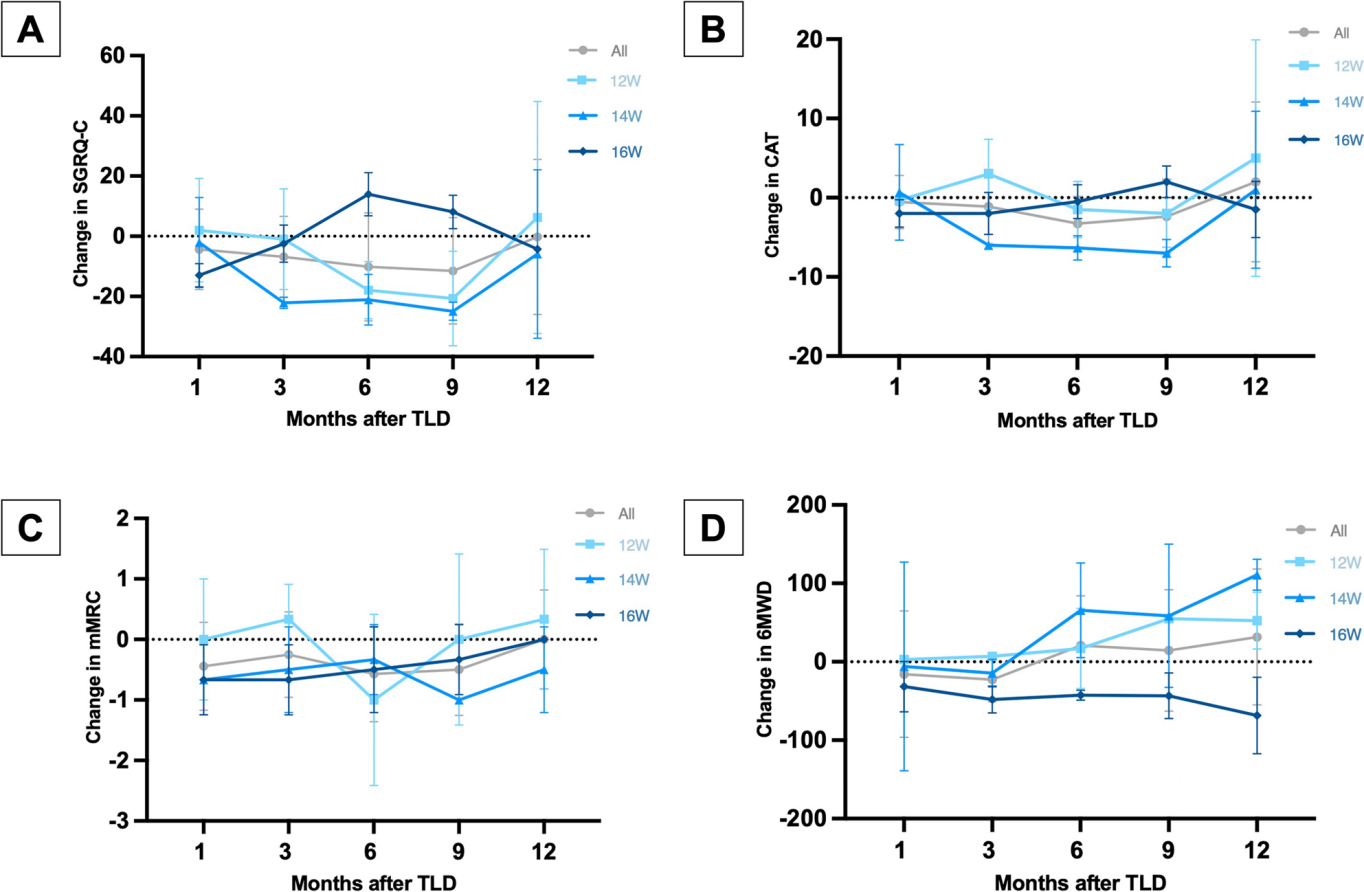
Relative change from baseline in quality-of-life scores SGRQ-C (**A**), CAT (**B**), mMRC (**C**), and exercise capacity 6WMD (**D**) after treatment in each group

As for the secondary clinical efficacy endpoints, the patients’ motor ability and quality of life scores showed improvement at the 6-month follow-up compared to baseline, with the 6MWD increasing by 21 ± 63 m; the SGRQ-C score decreasing by 13.55 ± 13.72 points; the mMRC score decreasing by 0.57 ± 0.79 points; and the CAT score decreasing by 3.29 ± 3.45 points. At the 12-month follow-up, the 6MWD increased 19 ± 86 m; the SGRQ-C score increased by 0.18 ± 23.93 points; the mMRC score decreased by 0.00 ± 0.76 points; the CAT score decreased by 3.00 ± 9.75 points. The absolute change results of motor ability and quality of life scores are shown in Table 4, and the relative changes are shown in Fig. 9.

**Table 4:** Absolute changes in exercise capacity assessment and health-quality of life compared to baseline after TLD treatment

	3 months	6 months	9 months	12 months
Exercise capacity assessment				
6MWD	-23 ± 28	21 ± 63	14 ± 77	19 ± 86
Health-quality of life				
SGRQ-C	-7.21 ± 14.49	-13.55 ± 13.72	-12.19 ± 17.56	0.18 ± 23.93
mMRC	-0.33 ± 0.71	-0.57 ± 0.79	-0.50 ± 0.76	0.00 ± 0.76
CAT	-1.44 ± 4.48	-3.29 ± 3.45	-2.38 ± 4.69	3.00 ± 9.75

## Discussion

In this study, we developed a novel TLD multi-polar RF ablation system and evaluated its safety, feasibility, and efficacy for moderate to severe COPD patients. It has been demonstrated in an in vitro lung model that irrigation of ice-cold saline at a high initial rate using a radiofrequency irrigation pump was shown to be effective in protecting the epithelial of the bronchial walls; and the depth required to achieve targeted ablation of pulmonary nerves can be achieved when the single electrode power is limited to 12–16 W in an in vitro liver model.

The safety and feasibility of this system have been preliminarily validated through preclinical animal experiments. Eight dogs were successfully treated with TLD without adverse events during follow-up. Sheep were selected for the next step in the validation of safety and efficacy because of their airway structural similarity to humans [11]. No significant adverse clinical effects or tissue damage near the ablation site were observed in the treated animals, and postprocedural pulmonary airway resistance was reduced by approximately 30% with a sustained 30% decrease in axonal staining, similar to published studies [10]. These results support our next first-in-human study. This first-in-man study is a dose-escalation study that confirms that TLD treatment with this novel RF ablation system is safe for COPD patients. The success rate of this instrument was 100% and the overall technical success rate was 88.9%. One case of procedural failure was caused by one of the electrodes failing to maintain adequate contact with the airway wall during the late ablation phase, resulting in high impedance and 54% of energy delivery. Notably, the ablation time was shorter than reported in previous studies due to the four electrodes in the ablation catheter. For efficacy, a trend of improvement in lung function, SGRQ-C, CAT, and 6MWD after TLD treatment was observed, suggesting the beneficial effects of TLD in COPD patients. Previous AIRFLOW-1trial reported a FEV_1_ and FVC improved at 1 year relative to baseline with average increases of 0.06 L and 0.2 L, respectively [16]. Subsequent AIRFLOW-2 trial reported an improvement at 1 year in FEV_1_ of 0.07 L and FVC of 0.2 L [13]. Recent AIRFLOW-3 trial reported both declines at 1 year in FEV_1_ of 0.01 L and FVC of 0.01 L [17]. According to our data, the overall population showed an improvement in FEV_1_ of 0.08 L and FVC of 0.3 L at 1-year post-TLD which is comparable to or even better than the previous studies. Although our study did not observe an improvement in QoL at 12 months, objective improvements in 6MWD and pulmonary function persisted. We believe this discrepancy may primarily be related to the small sample size of our study, as well as to the fact that QoL assessments are inherently subjective and relatively insensitive to clinically meaningful treatment effects in COPD [18]. Since the most significant improvements were demonstrated using a single-electrode ablative energy of 14 W, which is consistent with the preclinical findings, a larger-scale clinical study is currently being conducted using this energy level.

The highlight of this study is primarily the design of the multi-polar and adjustable circular part of the ablation catheter tip. This enables a loop bronchial denervation within a single energy delivery cycle, thereby shortening the TLD procedure time compared to the previous studies, and reducing the load and the technical requirements for the operators. Overall, multi-polar simultaneous ablation with a lower single electrode power can achieve a desired ablation effect, which avoids the risk of repeated ablations at the same site as well as reduces the damage to the airway tissue, which improves the generalizability and safety of the technique. In addition, the development of the ice-cold saline irrigation system showed the ability to reduce the temperature at the ablation site, resulting in less tissue damage. This study also has limitations. First, in the preclinical study, only the animals treated with 14 W were followed for 12 months; the long-term effects of higher energy have not been fully observed. Second, as the clinical study was designed as an exploratory trial, primarily to ensure the safety of the TLD procedures, it had some limitations, including a small cohort size and the absence of a sham-control group. Therefore, we are conducting a larger-scale clinical study to confirm the safety and efficacy of this technology in humans.

## Conclusion

We developed a novel multi-polar radiofrequency TLD system and demonstrated its feasibility and safety through in vitro and in vivo studies. The FIM clinical results confirmed the feasibility of this approach in humans, while its safety and efficacy warrant validation in larger patient cohorts.

## Supplementary Information

Supplementary Material 1. Supplementary Figure 1. Representative bronchoscopic images, histological alterations at bronchial ablation sites in sheep. (A1) Follow-up at 14/28 days post-TLD using 12W/14W/16W power. White arrows: granulation tissue. (A2) Long-term follow-up at 1/3/6/9/12 months post-14W TLD. HE staining demonstrated progressive inflammatory infiltration with increasing ablation power. Bronchial architecture was largely restored in the 12-month cohort. (B1) Axonal histology at 28 days post-TLD. Diffuse vacuolation throughout axonal regions (red arrows: pyknotic nuclei). (B2) Axonal histology at 12 months post-TLD showing attenuated vacuolation (red arrows: vacuoles/pyknotic nuclei). (B3-B4) Bronchial HE and Masson staining at 12 months post-TLD. Black arrows: axons; white arrows: fibrotic tissue.

Supplementary Material 2.

Supplementary Material 3.

Supplementary Material 4.

Supplementary Material 5.

Supplementary Material 6.

Supplementary Material 7.

## Data Availability

The datasets used and/or analysed during the current study are available from the corresponding author on reasonable request.
