# Supplementary figures and images for: A novel targeted lung denervation multi-polar radiofrequency ablation system for moderate to severe COPD patients: a translational study
Source: Respir Res. 2026 Jan 13;27:50. doi: 10.1186/s12931-026-03496-7 (PMC12888183; doi:10.1186/s12931-026-03496-7)

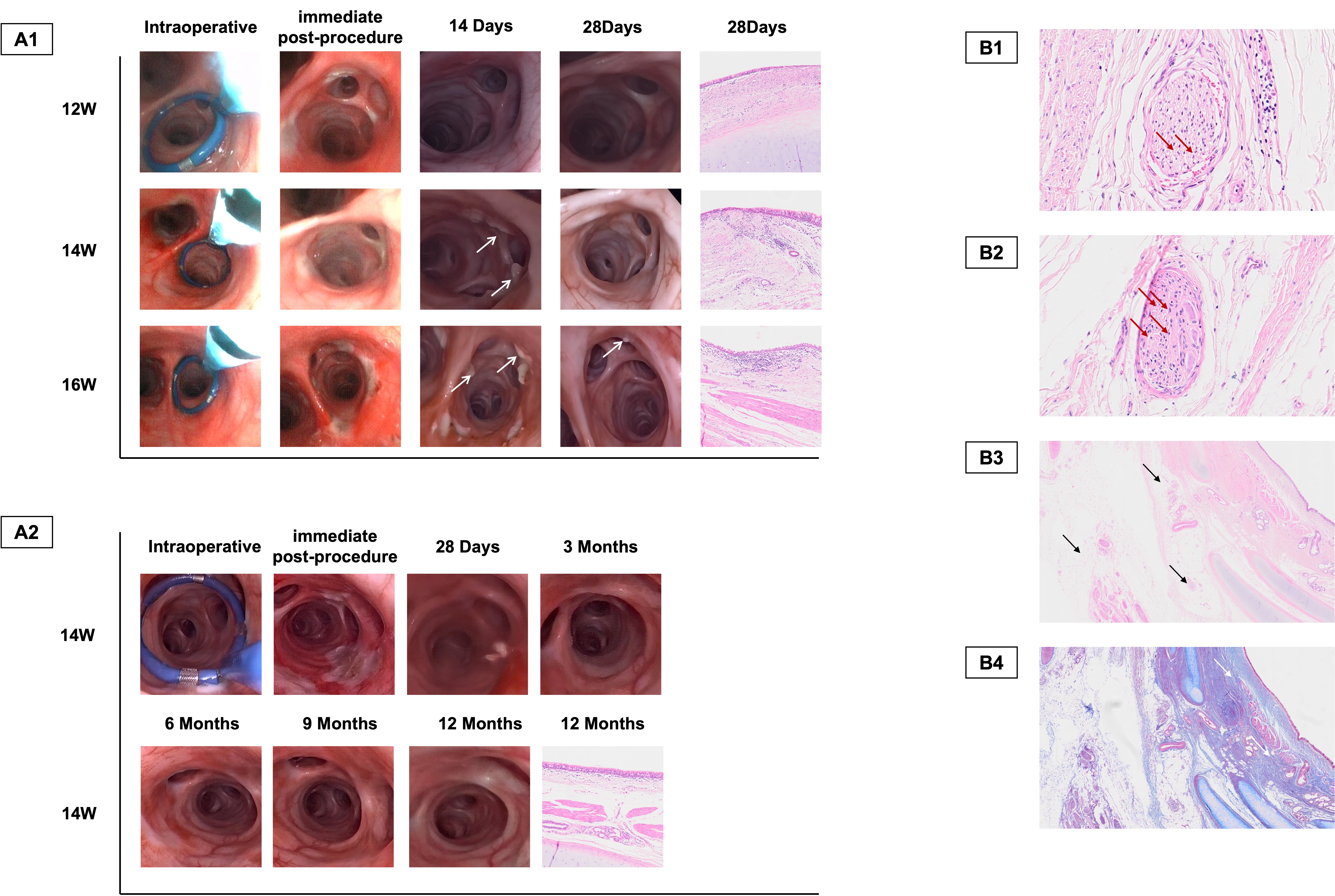

Supplement: Supplementary file 1 — Supplementary Material 1. Supplementary Figure 1. Representative bronchoscopic images, histological alterations at bronchial ablation sites in sheep. (A1) Follow-up at 14/28 days post-TLD using 12W/14W/16W power. White arrows: granulation tissue. (A2) Long-term follow-up at 1/3/6/9/12 months post-14W TLD. HE staining demonstrated progressive inflammatory infiltration with increasing ablation power. Bronchial architecture was largely restored in the 12-month cohort. (B1) Axonal histology at 28 days post-TLD. Diffuse vacuolation throughout axonal regions (red arrows: pyknotic nuclei). (B2) Axonal histology at 12 months post-TLD showing attenuated vacuolation (red arrows: vacuoles/pyknotic nuclei). (B3-B4) Bronchial HE and Masson staining at 12 months post-TLD. Black arrows: axons; white arrows: fibrotic tissue. [file 12931_2026_3496_MOESM1_ESM.jpg]
