# Supplementary material for: A novel targeted lung denervation multi-polar radiofrequency ablation system for moderate to severe COPD patients: a translational study
Source: Respir Res. 2026 Jan 13;27:50. doi: 10.1186/s12931-026-03496-7 (PMC12888183; doi:10.1186/s12931-026-03496-7)
Supplement: Supplementary file 2 — Supplementary Material 2. [file 12931_2026_3496_MOESM2_ESM.docx]

**Supplementary Table 1. Inclusion and exclusion criteria**

| Inclusion criteria | 1. Age ≥40 and≤ 75 years at the time of consent 2. Women with reproductive potential must undergo a negative pregnancy tests (serum or urine) at screening and no plans for pregnancy during the study period 3. Smoking cessation for a minimum of 2 months prior to consent and agrees to not smoke during the study; Participants using smoking cessation medications, patches, gum, etc., should undergo quantitative testing to assess whether nicotine or cotinine levels are below the study threshold 4. SpO_2_≥89% in room air at sea level during the screening 5. CAT≥10 or mMRC≥2 during the screening 6. Diagnosis of COPD according to GOLD 2021 guidelines, with 20%≤ FEV1% predicted <60% 7. Documented a history of taking ICS+LABA and LAMA+LABA as routine respiratory maintenance medications for ≥12 months at the time of informed consent 8. Patients considered suitable candidates for bronchoscopy based on investigator judgment (i.e., cardiovascular fitness, amenable to intubation, no previously diagnosed severe airway obstruction, and has no irreversible coagulopathy) 9. Willingness and ability to complete all baseline and follow-up assessments, including taking specific medications (e.g.azithromycin, prednisolone/ prednisone, etc) 10. The patient agrees to sign the informed consent form |
| --- | --- |
| Exclusion criteria | Exclusion Criteria:   1. BMI index <18 kg/m^2^ or >35 kg/m^2^ 2. Previously implanted electronic devices 3. Uncontrolled diabetes mellitus, such as HbA1c >7% 4. Presence of highly suspicious malignant lung nodules 5. Surgical, radiation, or chemotherapy treatment for malignant tumors within the 2 years preceding informed consent, as well as other thoracic surgeries 6. More than 3 respiratory-related hospitalizations in the year preceding informed consent 7. Diagnosed with asthma according to the current GINA guidelines 8. Previously diagnosed with non-COPD pulmonary diseases or with a history of pneumothorax 9. Clinically significant bronchiectasis, defined as severe single lobe or multilobar bronchial wall thickening and dilation observed on CT scans, results in persistent cough, intractable sputum, and repeated hemoptysis lasting for several days 10. Previously diagnosed with pulmonary arterial hypertension, defined as an estimated pulmonary artery systolic pressure >70mmHg on echocardiography 11. Myocardial infarction in the past 6 months, life-threatening arrhythmias or acute ischaemia indicated by electrocardiogram, Pre-existing evidence of ejection fraction <45%, or congestive heart failure of class C or D (ACC/AHA) or grade III or IV (NYHA) ,or any cardiovascular disease that prevents acceptance of a full anesthesia bronchoscopy 12. Known gastrointestinal motility disorders or previous abdominal surgeries involving the stomach, esophagus, or pancreas 13. Pre-treatment Gastroparesis Cardinal Symptom Index (GCSI) score ≥18.0 14. Patients with keloid diathesis 15. Patients with any diseases or conditions assessed by the investigator that may interfere with procedure or study completion 16. Prior daily use of >10mg prednisone or its equivalent before informed consent 17. Use of opioid medications in the 3 months preceding informed consent 18. Known contraindications or drug allergies to required bronchoscopy or general anesthesia that cannot be controlled with medication 19. Chest CT at screening showing bronchial anatomical structures preventing adequate treatment with existing catheters, such as adjacent severe bullae (>1/3 unilateral lung) or the discovery of a mass requiring treatment at the treatment site 20. Investigator assessment that the use of the targeted lung denervation system is technically impractical due to patient anatomy or other clinical findings 21. Patients are currently or planning to take medications affecting the M receptor mechanism for non-COPD-related diseases at the time of screening, such as male patients using M receptor blockers for prostate or bladder diseases, and are unable to cease usage for at least 1 day (short-acting drugs) or 7 days (long-acting drugs) before the planned procedure 22. Currently participating in other clinical trials |
| Withdrawal criteria | 1. The patient withdraws informed consent at any time 2. Onset of medical conditions where the investigator deems continued participation might compromise patient safety 3. The investigator judged that the suspension of the study was in the best interests of the patients 4. The patient does not comply with the study protocol |
