# Supplementary material for: A novel targeted lung denervation multi-polar radiofrequency ablation system for moderate to severe COPD patients: a translational study
Source: Respir Res. 2026 Jan 13;27:50. doi: 10.1186/s12931-026-03496-7 (PMC12888183; doi:10.1186/s12931-026-03496-7)
Supplement: Supplementary file 3 — Supplementary Material 3. [file 12931_2026_3496_MOESM3_ESM.docx]

**Supplementary Table 2. The ablation range with different single electrode powers**

| **Number of electrodes** | **Time of ablation/s** | **Ablation power of single electrode/W** | **Initial total impedance/Ω** | **Ablation range(mm)** |
| --- | --- | --- | --- | --- |
| 4 | 120 | 12 | 135 | 5.75±0.96 |
| 4 | 156 | 12 | 116 | 7±0.82 |
| 4 | 136 | 12 | 115 | 5.88±0.85 |
| 4 | 120 | 14 | 130 | 6.25±0.5 |
| 4 | 152 | 14 | 121 | 7.88±0.85 |
| 4 | 137 | 14 | 110 | 7.38±0.75 |
| 4 | 137 | 16 | 110 | 7.5±1.29 |
| 4 | 157 | 16 | 114 | 8.75±0.96 |
| 4 | 182 | 16 | 114 | 9.13±0.63 |
