# Supplementary material for: A novel targeted lung denervation multi-polar radiofrequency ablation system for moderate to severe COPD patients: a translational study
Source: Respir Res. 2026 Jan 13;27:50. doi: 10.1186/s12931-026-03496-7 (PMC12888183; doi:10.1186/s12931-026-03496-7)
Supplement: Supplementary file 4 — Supplementary Material 4. [file 12931_2026_3496_MOESM4_ESM.docx]

**Supplementary Table 3. The blood test of animals**

|  | **Before TLD** | **After TLD** | **7 Days** | **14 Days** | **21 Days** | **28 Days** | **3 months** | **6 months** | **9 months** | **12 months** |
| --- | --- | --- | --- | --- | --- | --- | --- | --- | --- | --- |
| WBC  (10^9^/L) | 8.14±1.81 | 6.97±1.22 | 9.89±1.18** | 6.88±1.23 | 8.26±1.40 | 7.36±2.71 | 4.82±1.01* | 5.66±1.61* | 4.83±1.77 | 4.19±0.81** |
| NEUT  (10^9^/L) | 4.58±1.19 | 3.85±1.06 | 4.52±0.71 | 3.38±0.50 | 3.06±0.57 | 4.19±2.36 | 2.26±0.43* | 3.41±1.73 | 2.86±1.54 | 2.58±0.68* |
| LYMPH  (10^9^/L) | 2.95±0.58 | 2.56±0.19 | 4.33±0.48** | 3.02±0.62 | 4.24±0.54* | 2.73±0.65 | 2.21±0.55 | 1.98±0.45* | 1.66±0.37** | 1.22±0.22** |
| MONO (10^9^/L) | 0.41±0.20 | 0.23±0.14 | 0.35±0.16 | 0.23±0.08 | 0.36±0.13 | 0.27±0.20 | 0.19±0.11 | 0.15±0.09 | 0.17±0.09 | 0.15±0.04 |
| EOS (10^9^/L) | 0.13±0.09 | 0.27±0.19* | 0.58±0.13** | 0.21±0.13 | 0.51±0.36* | 0.08±0.03 | 0.12±0.09 | 0.09±0.08 | 0.07±0.04 | 0.19±0.07 |
| BASO (10^9^/L | 0.03±0.01 | 0.03±0.01 | 0.05±0.02* | 0.02±0.01* | 0.04±0.01 | 0.02±0.01* | 0.02±0.01** | 0.01±0.00** | 0.01±0.01* | 0.01±0.01*** |
| LUC (10^9^/L | 0.04±0.03 | 0.03±0.03 | 0.07±0.04 | 0.03±0.02 | 0.05±0.04 | 0.07±0.12 | 0.04±0.02 | 0.03±0.02 | 0.06±0.04 | 0.04±0.01 |
| NEUT% (%) | 56.02±5.55 | 52.82±6.69** | 45.52±2.64* | 49.38±2.61 | 37.00±2.13** | 54.70±9.63 | 47.06±3.79** | 57.96±12.21 | 57.46±9.56 | 60.96±5.38 |
| LYMPH  (%) | 36.54±4.07 | 37.54±7.30 | 43.84±2.56 | 43.88±3.41* | 51.68±4.00** | 39.68±11.08 | 45.66±3.71** | 37.06±11.81 | 36.20±9.44 | 29.68±5.46* |
| MONO%(%) | 5.08±1.86 | 4.00±2.43 | 3.46±1.28 | 3.22±0.88 | 4.42±1.47 | 3.40±1.07 | 3.70±1.66 | 2.62±1.73 | 3.50±1.55 | 3.72±1.56 |
| EOS% (%) | 1.54±0.78 | 4.60±2.84* | 5.94±1.71** | 2.96±1.53 | 5.86±3.78* | 1.16±0.36 | 2.48±1.68 | 1.70±1.53 | 1.46±0.82 | 4.50±0.89** |
| BASO% (%) | 0.36±0.15 | 0.44±0.13 | 0.52±0.15 | 0.22±0.08 | 0.44±0.15 | 0.26±0.15 | 0.30±0.10 | 0.18±0.04* | 0.16±0.09* | 0.16±0.11** |
| LUC% (%) | 0.48±0.22 | 0.60±0.45 | 0.68±0.41 | 0.40±0.29 | 0.62±0.51 | 0.80±0.93 | 0.82±0.46 | 0.50±0.37 | 1.18±0.37 | 1.02±0.33* |
| RBC (10^12^/L) | 12.71±1.48 | 9.50±2.05 | 12.82±2.10 | 11.53±1.44 | 12.17±1.84 | 12.12±1.69 | 11.27±1.47 | 11.49±0.77 | 10.17±0.53* | 8.44±1.08** |
| HGB  (g/L) | 131.20±20.32 | 102.00±16.14 | 129.60±12.30 | 120.40±10.83 | 125.80±12.79 | 128.20±13.70 | 127.60±6.69 | 128.40±10.36 | 120.00±10.37 | 101.00±8.15* |
| HCT  (%) | 37.20±5.64 | 28.02±4.18 | 36.72±3.01 | 34.44±2.74 | 35.64±2.49 | 37.20±3.71 | 35.92±2.18 | 36.96±3.15 | 34.36±3.00 | 28.06±2.97* |
| MCV  (fL) | 29.22±1.60 | 30.00±3.02 | 29.06±3.30 | 30.12±2.85 | 29.64±3.26 | 30.90±2.79* | 32.17±3.31* | 32.14±1.69** | 33.78±1.68*** | 33.34±1.82** |
| MCH  (pg) | 10.30±0.59 | 10.90±0.94 | 10.24±0.90 | 10.54±0.89 | 10.42±0.82 | 10.62±0.82 | 11.44±1.16** | 11.18±0.49*** | 11.80±0.52*** | 12.02±0.86** |
| MCHC (g/L) | 353.00±3.46 | 363.80±11.43 | 352.80±13.29 | 349.20±5.81 | 352.60±12.54 | 344.40±5.22* | 355.60±4.56 | 347.60±8.23 | 348.80±6.22 | 361.00±10.86 |
| RDW  (%) | 16.48±0.66 | 17.00±0.93 | 18.02±1.94 | 17.16±0.38* | 17.26±0.52 | 17.30±0.58* | 18.28±1.20* | 17.10±1.16 | 16.56±1.13 | 16.76±0.44 |
| PLT  (10^9^/L) | 678.20±163.92 | 560.00±152.05 | 719.80±234.95 | 548.60±135.51 | 582.20±230.55 | 436.60±133.53* | 375.20±154.24 | 464.80±135.50** | 281.40±136.90*** | 298.00±209.94** |
| MPV  (fL) | 8.16±0.37 | 8.28±1.82 | 8.40±1.32 | 7.92±0.69 | 7.38±0.86 | 9.62±0.91* | 10.14±2.13 | 8.28±0.79 | 8.18±2.13 | 10.66±2.93 |
| RETIC (10^12^/L) | 0.01±0.00 | 0.02±0.02 | 0.01±0.00 | 0.02±0.00** | 0.02±0.01** | 0.01±0.00 | 0.02±0.02 | 0.01±0.00 | 0.01±0.01 | 0.01±0.00* |
| RETIC% (%) | 0.08±0.03 | 0.26±0.29 | 0.07±0.03 | 0.17±0.05*** | 0.15±0.04** | 0.10±0.03 | 0.16±0.19 | 0.12±0.04 | 0.15±0.07* | 0.17±0.02** |
| ALTL (U/L) | 7.50±2.05 | 7.32±1.84 | 10.94±3.44* | 9.44±3.08 | 9.86±2.30 | 7.76±3.76 | 12.66±2.20 | 10.62±4.80 | 9.94±2.36 | 12.30±6.67 |
| ASTL (U/L) | 89.96±24.18 | 73.32±25.27 | 64.54±35.38 | 65.94±15.00 | 50.06±25.76* | 92.40±15.98 | 97.26±48.94 | 92.26±30.61 | 91.12±20.23 | 183.60±85.51 |
| CREJ2 (μmol/L) | 87.40±15.04 | 89.00±9.95 | 46.00±15.91** | 67.40±13.41** | 54.60±19.37* | 60.80±25.69 | 67.40±6.95 | 84.40±12.28 | 90.60±20.67 | 72.60±7.70 |
| GGTI2 (U/L) | 61.42±25.12 | 53.84±5.07 | 49.84±12.11 | 56.78±9.51 | 80.46±132.75 | 56.12±6.47 | 52.90±30.86 | 74.14±11.65 | 77.58±7.71 | 96.90±23.12** |
| U-BUN (mg/dL) | 14.96±12.33 | 24.44±5.44 | 14.20±5.46 | 18.44±7.61 | 9.02±7.91 | 16.62±9.08 | 15.02±8.57 | 15.66±3.99 | 14.90±3.99 | 19.66±3.66 |
