# Supplementary material for: A novel targeted lung denervation multi-polar radiofrequency ablation system for moderate to severe COPD patients: a translational study
Source: Respir Res. 2026 Jan 13;27:50. doi: 10.1186/s12931-026-03496-7 (PMC12888183; doi:10.1186/s12931-026-03496-7)
Supplement: Supplementary file 5 — Supplementary Material 5. [file 12931_2026_3496_MOESM5_ESM.docx]

**Supplementary Table 4. Treatment-related adverse events within 6 months following TLD treatment.**

| **MedDRA System organ class (SOC)**  **Preferred term （PT）** | **Number（N=9）**  **n（%）** | **Frequency**  **(n=33)** |
| --- | --- | --- |
| **Respiratory tract, thoracic cavity and mediastinum disease** | 5（55.5） | 28 |
| Hemoptysis | 2（22.2） | 2 |
| Cough | 2（22.2） | 3 |
| Expectoration | 1（11.1） | 3 |
| Larynx pain | 1（11.1） | 1 |
| Pharynx pain | 1（11.1） | 1 |
| Dyspnea | 1（11.1） | 3 |
| Chronic obstructive pulmonary disease * | 3（33.3） | 4 |
| Upper respiratory tract infection | 1（11.1） | 2 |
| Lower respiratory tract infection | 1（11.1） | 1 |
| Infective pneumonia | 1（11.1） | 1 |
| Chest discomfort | 1（11.1） | 1 |
| Wheezing | 1（11.1） | 1 |
| Chest pain | 3（33.3） | 5 |
| **Systemic disease and various reactions at the administration site** | 1（11.1） | 2 |
| Weakness | 1（11.1） | 1 |
| Fatigue | 1（11.1） | 1 |
| **Gastrointestinal system disease** | 1（11.1） | 1 |
| Abdominal distension | 1（11.1） | 1 |
| **Musculoskeletal and connective tissue disease** |  |  |
| Backache | 1（11.1） | 2 |

* refers to acute exacerbation of chronic obstructive pulmonary disease (AECOPD).
