# Supplementary material for: A novel targeted lung denervation multi-polar radiofrequency ablation system for moderate to severe COPD patients: a translational study
Source: Respir Res. 2026 Jan 13;27:50. doi: 10.1186/s12931-026-03496-7 (PMC12888183; doi:10.1186/s12931-026-03496-7)
Supplement: Supplementary file 7 — Supplementary Material 7. [file 12931_2026_3496_MOESM7_ESM.docx]

**Supplementary Table 6. GCSI score within 12 months following TLD treatment.**

| No. | GCSI 0M | GCSI 3M | GCSI 6M | GCSI 9M | GCSI 12M | △GCSI 3M | △GCSI 6M | △GCSI 9M | △GCSI 12M |
| --- | --- | --- | --- | --- | --- | --- | --- | --- | --- |
| 001 | 3 | 3 | 0 | 1 | 1 | 0 | -3 | -2 | -2 |
| 002 | 0 | 3 | 1 | 0 | 0 | 3 | 1 | 0 | 0 |
| 003 | 0 | 0 | NA | NA | 9 | 0 | NA | NA | 9 |
| 004 | 0 | 1 | 1 | 0 | 0 | 1 | 1 | 0 | 0 |
| 005 | 2 | NA | 2 | 0 | 1 | NA | 0 | -2 | -1 |
| 006 | 2 | 8 | 2 | 6 | NA | 6 | 0 | 4 | NA |
| 007 | 0 | 0 | 0 | 0 | 0 | 0 | 0 | 0 | 0 |
| 008 | 0 | 0 | 0 | 0 | 0 | 0 | 0 | 0 | 0 |
| 009 | 0 | 3 | NA | 0 | 2 | 3 | NA | 0 | 2 |
